# Development and validation of a brief general and sports nutrition knowledge questionnaire and assessment of athletes’ nutrition knowledge

**DOI:** 10.1186/s12970-018-0223-1

**Published:** 2018-04-19

**Authors:** Gina Louise Trakman, Adrienne Forsyth, Russell Hoye, Regina Belski

**Affiliations:** 1 Department of Rehabilitation, Nutrition and Sport, School of Allied Health, College of Science, Health and Engineering, La Trobe University, Melbourne, 3086 Australia; 2 Office of the Deputy Vice-Chancellor Research, La Trobe University, Melbourne, 3086 Australia; 3 Department of Health Professions, School of Health Science, Faculty of Health, Art and Design, Swinburne University of Technology, Melbourne, 3122 Australia

**Keywords:** Sport, Nutrition knowledge, Knowledge, Food, Diet, Athlete, Questionnaires, Response rate, Completion rate

## Abstract

**Background:**

The Nutrition for Sport Knowledge Questionnaire (NSKQ) is an 89-item, valid and reliable measure of sports nutrition knowledge (SNK). It takes 25 min to complete and has been subject to low completion and response rates. The aim of this study was to develop an abridged version of the NSKQ (A-NSKQ) and compare response rates, completion rates and NK scores of the NSKQ and A-NSKQ.

**Methods:**

Rasch analysis was used for the questionnaire validation. The sample (*n* = 181) was the same sample that was used in the validation of the full-length NSKQ. Construct validity was assessed using the known-group comparisons method. Temporal stability was assessed using the test-retest reliability method. NK assessment was cross-sectional; responses were collected electronically from members of one non-elite Australian football (AF) and netball club, using Qualtrics Software (Qualtrics, Provo, UT).

**Results:**

*Validation -* The A-NSKQ has 37 items that assess general (*n* = 17) and sports (*n* = 20) nutrition knowledge (NK). Both sections are unidimensional (Perc5% = 2.84% [general] and 3.41% [sport]). Both sections fit the Rasch Model (overall-interaction statistic mean (SD) = − 0.15 ± 0.96 [general] and 0.22 ± 1.11 [sport]; overall-person interaction statistic mean (SD) = − 0.11 ± 0.61 [general] and 0.08 ± 0.73 [sport]; Chi-Square probability = 0.308 [general] and 0.283 [sport]). Test-retest reliability was confirmed (*r* = 0.8, *P* < 0.001 [general] and *r* = 0.7, P < 0.001 [sport]). Construct validity was demonstrated (nutrition students = 77% versus non-nutrition students = 60%, P < 0.001 [general] and nutrition students = 60% versus non-nutrition students = 40%, P < 0.001 [sport]. *Assessment of NK -* 177 usable survey responses from were returned. Response rates were low (7%) but completion rates were high (85%). NK scores on the A-NSKQ (46%) are comparable to results obtained in similar cohorts on the NSKQ (49%). The A-NSKQ took on average 12 min to complete, which is around half the time taken to complete the NSKQ (25 min).

**Conclusions:**

The A-NSKQ is a valid and reliable, brief questionnaire designed to assess general NK (GNK) and SNK.

**Electronic supplementary material:**

The online version of this article (10.1186/s12970-018-0223-1) contains supplementary material, which is available to authorized users.

## Background

Nutrition knowledge (NK) is a modifiable determinant of dietary behaviour [1, 2] and dietary practices are known to influence athletic performance [3]. Therefore, there has been much interest in the assessment of athletes’ general nutrition knowledge (GNK) and sports nutrition knowledge (SNK) [4, 5]. There are limitations with some of the NK questionnaires that have previously been employed, including testing outdated recommendations, lack of comprehensiveness, lack of cultural appropriateness, and insufficient validation [6]. The issue of inadequate validation is common in evaluation measures used in nutrition education research [7]. Researchers may overlook comprehensive methodologies because the time taken to develop and validate questionnaires can be prohibitive [8].

The Nutrition for Sport Knowledge Questionnaire (NSKQ) [9] was developed in 2017 and overcame many of the aforementioned limitations. The NSKQ was based on current sports nutrition recommendations [10, 11] and is comprehensive, containing 89 questions across six nutrition sub-sections. Moreover, it was developed with a panel of international sports dietitians and validated using a robust methodology that included both Classical Test Theory (CTT) [8] and Rasch analysis [12].

Other recently developed SNK questionnaires also represent an improvement on previous tools and are of similar length to the NSKQ [13–15]. When collecting data to assess the validity of the NSKQ [9], and (later) when using the NKSQ to evaluate Australian Footballers’ NK 1 response and completion rates were low. The NSKQ takes athletes on average 25 min to complete. Some researchers have reported that the ideal questionnaire completion time to optimise response rates is 13 min or less [16]. Galesic et al. [17] reported that, in general, online-questionnaires that were perceived to be long were started and completed by fewer respondents, with less time spent on questions at the end of questionnaires. In contrast, a meta-analysis of factors influencing response rates of online-questionnaires found that questionnaire length had a very small effect (*r* = 0.001) on response rates [18], but the paper did not report on completion rates.

To our knowledge, there is no published data specifically on the effect of questionnaire length on response and completion rates in athletic cohorts. Both elite and non-elite sportspeople are often pressed for time, balancing busy training schedules with other commitments [19]. Therefore, it is feasible that long questionnaires would be daunting to athletes and the professionals working with them, and in part, explain the low response and completion rates of the NSKQ.

The aforementioned factors demonstrate a potential need for shorter measurement tools to assess NK amongst athletes. Appropriate validation of such tools is critical if they are to be used by dietitians and coaches with the intention of influencing athlete behaviour and ultimately performance. Rasch analysis is a method which allows researchers to produce short measurement tools that include both difficult and simple items, and is therefore a suitable method for use in developing a brief NK questionnaire [20]. The Rasch model presumes individuals with higher levels of knowledge are more likely to obtain a high NK score and that easy items are more likely to be answered correctly by all respondents [20]. This is advantageous because it means the focus of validation is on the questionnaire as a whole, rather than on individual items [21].

The aims of this study were to:

1. re-assess data used to validate the NSKQ to develop an abridged version of the questionnaire (the A-NSKQ) and
2. compare and contrast response rates, completion rates and NK scores between NSKQ and A-NSKQ in a cohort of non-elite AF and netball athletes.

It was hypothesised that the A-NSKQ would achieve higher completion rates than the NSKQ, and produce NK scores that are comparable to a related cohort (non-elite AF players).

## Methods

### Institutional review board

The research was approved (S16/267) by the La Trobe University’s SHE College Human Ethics Sub-Committee (SHE CHESC). All participants were provided with the participant information statement and consent form (online) and ‘agreed’ to participate (electronically).

### Instrument development

The data used to develop the abridged version of the NSKQ was the same data that was collected for the final stages of the full-length NSKQ validation. Participants (*n* = 181) were Australian athletes and university students, recruited from November 2016 to January 2017 [9]. The sample was predominately female (75%), aged 17–25 (52%), born in Australia (81%), and university educated (96%) (Additional file 1: Table S1).

A detailed description of the development and validation of the full-length NSKQ are described elsewhere [9]. The full-length NSKQ was developed in accordance with methods recommended by Trakman et al. [6], and the validation of the A-NSKQ was based on a modified version of these methods. In the validation of the A-NSKQ no new items were developed (steps 1–3) and face and content validity (steps 4 and 5) were not re-assessed (Table 1).

**Table 1:** Comparison of methods used in the development and validation of the NSKQ and A-NSKQ

NSKQ development and validation	A-NSKQ validation
Development of the tool: 1. Define construct and develop test plan. 2. Generate items. 3. Choose scoring system **Preliminary review of the items:** 4. Assess content validity. 5. Asses face validity Further statistical analysis of measurement: 6. Purification of the scale using item analysis 7. Evaluate internal reliability and use of Rash analysis to assess item/person indicators, dimensionality and internal reliability **Final analysis**: 8. Re-examine questionnaire properties, assess temporal stability and confirm construct validity.	**Using data from step (8)** 1. Rash analysis to assess item/person indicators, dimensionality and internal reliability 2. Assess temporal stability and construct validity, using proxy-scores.

Item analysis (step 6) was performed using Rasch analysis only. Rasch analysis was conducted using RUMM2030 Professional software. The aim of this step was to remove items that were causing misfit to the Rasch model. Misfit was assessed based on overall item-interaction, overall person-interaction and overall item-trait interaction. A SD of 1 and mean of 0 for overall-item and overall-person interaction, and non-significant Chi-Square probability for overall item-trait interaction indicate compliance with the Rasch model (i.e. no misfit). In order to determine which items (or persons) were causing misfit, the following indicators were evaluated: individual item fit residuals, item characteristic curves (ICC), category probability curves (CPC) and differential item functioning (DIF). Detailed definitions and interpretation of the aforementioned statistics are beyond the scope of this report, but are described elsewhere [6, 12] and summarised in Additional file 1: Table S2. Stricter criteria were applied during item analysis for validation of the A-NSKQ. During the validation of the full-length NSKQ the researchers retained certain items that did not meet one or two of the aforementioned Rasch indicators because they were deemed relevant in terms of assessing gaps in NK and retaining content validity. However, in the validation of the A-NSKQ, all items that failed on a single indicator were excluded.

Assessment of internal reliability and dimensionality (step 7) were also performed using Rasch analysis only for the validation of the A-NSKQ. Reliability was assessed using the PerSepIndex, a summary statistic produced by RUMM 2030 that is analogous to Cronbach α. Both PerSepIndex and Cα are based on repeated split-half reliability assessment; a value of ≥0.7 is an acceptable measure of internal reliability [12]. Dimensionality was assessed using the perc5% statistic; values of 5% or less indicate unidimensionality, a requirement of the Rasch model [20].

Gathering of new data to re-examine the scale (step 8) was not conducted. Rather, a ‘proxy score’ for each respondent was calculated by subtracting the deleted NSKQ items from participants’ original score. Similar methods were used by past researchers [22] to compare scores on the original and revised General Nutrition Knowledge Questionnaire (GNKQ/R-GNKQ). Using the proxy scores, construct validity was assessed using the known-group comparisons method and temporal stability was assessed using the test-retest reliability method (Table 1). For the known-group comparisons assessment, NK (proxy) scores of individuals with and without a formal nutrition education (university course, university subject, diploma, and online course) were compared; statistically significant differences are indicative of construct validity [6]. For the test-retest reliability assessment the correlation of NK (proxy) scores on two attempts of the questionnaire, taken ten days to two weeks apart, were assessed; a correlation of ≥0.7 indicates temporal stability [6]. The correlation between NK proxy scores and original scores was also assessed.

### Athletes’ nutrition knowledge

After the NSKQ had been revised and the A-NSKQ had been validated, it was administered to Australian non-elite athletes. Members (*n* = 3951) of one metropolitan AF and netball league in Melbourne, Australia were invited (via email from the league president) to participate in the study by completing the A-NSKQ online. Interested and eligible (residing in Australia, aged 17 years and older) players completed the survey using Qualtrics Research Suite (Qualtrics, Provo, Utah) from May to April 2017.

### Data analysis

All analyses (aside from questionnaire validation) were performed in SPSS (Version 23; SPSS, Chicago, IL, USA). Scores on the A-NSKQ and time taken to complete the A-NSKQ were assessed for normality using the Kolmogorov-Simonov statistic. Chi-Square goodness of fit tests were used to evaluate differences in (categorical) demographic characteristics across gender, sport played, highest level of education, previous history of nutrition education, and highest level sport played. Differences in NK scores were assessed using t-test or ANOVA for parametric data, and Mann-Whitney-U-test or Kruskal-Wallis for non-normal data. Significant differences in questionnaire sub-section scores were assessed using paired sample t-test or Wilcoxon-signed-rank-test. The alpha value for most tests was set at *P* ≤ 0.05. A Bonferroni correction was applied to Kruskal-Wallis tests, and P was set at ≤0.017. Respondents with more than 10% missing data were removed from analysis. Missing responses to items were coded as incorrect.

## Results

### Instrument development

The original NSKQ consisted of 89 items. Rasch analysis showed that three items had high fit residuals, 19 items had problematic CPCs, 38 items had problematic ICCs, and one item showed DIF (for country). Nine items failed on two indicators, therefore, in total, 52 items were problematic and were excluded from the A-NSKQ, leading to a 37-item questionnaire.

The A-NSKQ was not unidimensional (Perc5% = 7.39%). The two sub-sets of items that were most different from each other, based on principal components analysis (PCA), were not theoretically related. Therefore, the investigator (GT) divided the items into two sub-sections based on their content. The first sub-section (‘general nutrition knowledge’) included 17 items that assessed knowledge of energy density; the role and sources of macronutrients and micronutrients; and alcohol. The second section (‘sports nutrition knowledge’) included 20 items that assessed knowledge of athletes’ macronutrient and fluid requirements; weight loss and gain strategies for athletes; and supplementation for athletes. These two sub-sections fit the Rasch model and were unidimensional. Reliability of the whole scale (PerSepIndex = 0.8) and the SNK (PerSepIndex = 0.7) were acceptable; the value for the GNK (PerSepIndex =0.6) was below the requisite value of 0.7 (Table 2).

**Table 2:** Summary of properties of the A-NSKQ

Section (number of questions)	Overall-item interaction statistics† (Mean ± SD)	Overall-person interaction statistics † Mean (SD)	Chi-Square probability ‡ (*P*-value)	Perc5% ¶	Person Separation Index ¥	Test-retest reliability ¥ (Pearson’s r)	Score (%)
Total (*n* = 37)	0.04 ± 0.90	0.01 ± 0.74	0.076	7.39	0.8	0.7*	Nutrition: 66 +/−14 § Non-nutrition: 52+/−13*
General (*n* = 17)	−0.15 ± 0.96	−0.11 ± 0.61	0.308	2.84	0.6	0.8*	Nutrition: 77 (24) ǁ Non-nutrition: 65 (25) *
Sports (*n* = 20)	0.22 ± 1.11	−0.08 ± 0.73	0.283	3.41	0.7	0.7*	**ǁ** Nutrition: 60 (25) Non-nutrition: 40 (21)*

† Mean ± SD = 0 ± 1 = perfect fit; SD > 1.5 = misfit. ‡Non-significant *p*-value = fit. ¶ < 5% = unidimensionality. ¥ PerSepIndex> 0.7 = adequate internal reliability. ¥ *r* ≥ 0.7 = test-retest reliability. § Score reported as mean **+/−** SD. ǁ score reported as median (IQR) * *P* = < 0.001

Individuals who had undertaken nutrition studies scored better in the GNK and SNK sub-sections (nutrition students = 77% versus non-nutrition students = 60%, *P* < 0.001 [GNK] and nutrition students = 60% versus non-nutrition students = 40%, P < 0.001 [SNK]), indicating construct validity. Test re-test reliability was demonstrated based on the proxy scores (*r* = 0.8, *P* < 0.001 [GNK] and *r* = 0.7, *P* < 0.001 [SNK]). There was a strong, positive correlation (*r* = 0.9, P < 0.001) between individuals’ score (%) on the NSKQ and their proxy score on the A-NSKQ. This indicates the removal of the items did not alter overall total (%) NK scores.

### A-NSKQ response and completion rates

Two-hundred-and-seventy-three athletes followed the link to complete the A-NSKQ (response rate = 7%). The individual response rate of the NSKQ could not be calculated for non-elite athletes, because the questionnaire was distributed by a second party and the total number of invitations was not known; the response rate for team presidents who agreed to distribute the NSKQ was 8–13% (Additional file 1: Table S2).

After deleting responses of individuals who followed the link but did not agree to the participant information and consent form (*n* = 65; not included in completion rate calculations), incomplete responses (*n* = 22) and respondents who did not meet the eligibility criteria for age (*n* = 9), there were 177 usable responses. The completion rate for the A-NSKQ (85%) was higher than the average completion rate for the NSKQ (54%); completion rate calculations were undertaken in the same manner (Additional file 1: Table S2).

The median time taken to complete the A-NSKQ was 12 min, compared to 25 min taken to complete the NSKQ.

### A-NSKQ participant characteristics

The characteristics of the participants who completed the A-NSKQ are outlined in Table 3. The sample was predominately female (61%) and aged 17–25 years (48%). Despite being distributed to the database of a recreational AF and netball league, 23% of athletes stated the main sport they played was not AF or netball, and 19% of athletes reported competing at the state, national or international level.

**Table 3:** Participant characteristics of individuals who completed the A-NSKQ

Age, n (%)	
17–25	85 (48)
26–35	66 (37)
≥ 36	26 (15)
Gender, n (%)	
Male	69 (39)
Female	108 (61)
Country of Birth, n (%)	
Australia	167 (94)
Outside Australia	10 (6)
Highest Level of Education, n (%)	
High School	32 (18)
Diploma	28 (16)
University	117 (66)
Formal Nutrition Studies, n (%)	
Yes	37 (21)
No	140 (79)
Sport Played, n (%)	
AF	129 (73)
Netball	8 (5)
Other	40 (23)
Highest level of sport played, n (%)	
Metropolitan (Non-elite)	144 (81)
State (Non-elite)	25 (14)
National (Elite)	6 (3)
International (Elite)	2 (1)
Years Playing Sport, Median (IQR)	9.5 (12.0)
Hours Training, Median (IQR)	7.0 (5.0)
BMI, Median (IQR)	23.9 (4.5)

There were no differences in age, gender, country of birth, level of education and previous nutrition study between those who reported playing at the elite and non-elite level. However, individuals who reported playing a sport other than AF as their primary sport were more likely to play at higher levels of competition (*X*^*2*^ = 104.526, df = 1, *P* < 0.001). Females were more likely to play a sport other than AF (*X*^*2*^ = 4.637, df = 1, *P* = 0.031) and to be university educated (*X*^*2*^ = 0.337, df = 1. P < 0.001).

### Nutrition knowledge of athletes

#### Knowledge scores

The mean total score was 47 ± 12%. There was a large variability amongst participants and between SNK and GNK (Table 4).

**Table 4:** Scores on the A-NSKQ

Section	Total Score (%)	Minimum Score (%)	Maximum Score (%)
Total	†47 ± 12	8	78
GNK	‡59 (18)*	18	82
SNK	‡35 (18)*	0	70

* *P* < 0.001 § reported as mean ± SD ǁ reported as median

#### Responses to individual items and gaps in knowledge

Several misconceptions were evident, especially with regards to hydration, micronutrients and supplementation. For example, only 8% of athletes knew that they should drink to maintain plasma volume and 94% thought that vitamin B1 was needed for delivery of oxygen to tissues’. In contrast, questions on the role and sources of fat, the effect of alcohol on performance and post-exercise snacks were answered correctly by more than 70% of participants (Additional file 1: Table S4).

#### Individual characteristics and knowledge

Stratified results based on individual characteristics are reported in Additional file 1: Table S5. Individuals with a formal nutrition education scored significantly higher in both the SNK (*P* = 0.003) and GNK (*P* = 0.003) section. There was a significant difference in SNK (*P* = 0.037) and GNK (*P* = 0.003) based on level of education. For GNK and SNK, when a Bonferroni correction was applied, only the difference between high school and diploma was statistically significant; *P* = 0.013 [GNK] and (*P* = 0.0011)[SNK]. There was also a significant difference in GNK based on age (*P* = 0.013). Individuals aged ≥36 scored statistically significantly higher than those aged 17–25 (*P* = 0.004) and those aged 26–35 (*P* = 0.003).

## Discussion

The aims of this study were to (1) re-assess data used to validate the NSKQ to develop an abridged version of the questionnaire (the A-NSKQ) and (2) compare and contrast response rates, completion rates and NK scores between NSKQ and A-NSKQ in a cohort of non-elite AF and netball athletes. The authors hypothesized that the A-NSKQ would achieve similar results to, but higher completion rates than, the NSKQ.

### The instrument

The 37-item A-NSKQ can be completed in around half the time taken to complete the NSKQ (12 versus 25 min). The A-NSKQ covers the same key topics assessed in the NSKQ (weight management, macronutrients, micronutrients, supplementation, sport nutrition, and alcohol);however, it does not evaluate some aspects of these topics, such as the role of specific supplements, and the consequences of dehydration. Therefore, the content and face validity of the A-NSKQ should be reviewed by individuals planning on using the tool, to ensure that it serves their intended purpose.

The results of this study show that the A-NSKQ sub-sections (GNK and SNK) fit the Rasch model, indicating that the items meet the requirements that difficult questions are less likely to be answered correctly and individuals with higher levels of NK are more likely to perform well [20]. Likewise, the questionnaire demonstrated test-retest reliability and achieved or approached pre-determined cut-off values for internal reliability. However, a limitation is that these assessments were based on proxy scores. Construct validity was also determined based on the proxy scores, and was confirmed when the A-NSKQ was administered to non-elite athletes, as those with previous nutrition education scored significantly higher than those without any formal nutrition education (Additional file 1: Table S5). The criterion validity of the A-NKSQ could be assessed in future studies by administrating both questionnaires to participants and calculating the correlation between scores (%). However, it may be difficult to achieve adequate response rates for this step, as respondents are likely to find completing both tools burdensome. In addition, participants may research the correct answers to questions before the second completion, which would influence the validity of this method.

### Response and completion rates

The invitation to complete the A-NSKQ was distributed to a large number of non-elite athletes via email, but response rates were low (7%). Although web-surveys are known to have lower response rates than mail-based surveys, these usually sit around 34% [23]. Response rates for NSKQ could not be calculated because it was distributed via Facebook groups and online forums, or via second parties where total exposure was not known. However, the percentage of non-elite teams who responded positively to be involved in data collection using the NSKQ (6–13%) was very similar to the response rates of individuals who participated in data collection using the A-NSKQ (7%). This is in line with some reports in the literature that questionnaire length has only a small effect on response rates [18]. Although the shorter length did not appear to have a positive effect on response rates, it did have a positive effect on completion rates. Completion rates in the present study were 85%, which is higher than completion rates (54%) achieved during the validation and use of NSKQ to collect data. As above, this finding is akin to what has previously been reported regarding questionnaire length and completion rates [17]. Future studies should consider distribution methods and survey design to ensure that response and completion rates are optimised. These include designing personalised invitations and sending reminders and notifications if responses have not been obtained [16]. However, personal interest in the topic is one of the key factors influencing individuals’ decision to participate in a survey, and this cannot be controlled by investigators [16].

### Nutrition knowledge of athletes

The results indicate there is room for improvement in non-elite AF and netball players’ NK. Athletes’ mean score was 47%; similarly, non-elite, male AF players scored 51% on the NSKQ ^1^. In the present study, athletes performed much better on questions of GNK than SNK, which is akin to results reported by Devlin et al. [24] and Kunkel et al. [25], but in contrast to results reported by Harrison et al. [26] and Barr [27]. Respondents answered items on hydration (q27) supplements (q35, q26, and q37) and micronutrients (q13, q 23, and q25) poorly. These items were also answered poorly when the NSKQ was administered to non-elite AF players^1^. In the existing literature there are inconsistencies regarding gaps in knowledge with some studies reporting hydration [28], supplementation [29] and micronutrients [28] were better understood than other topics, and others finding the opposite to be true [26, 30].

A literature review undertaken by Trakman et al. [31] reported that 64% of studies on athletes’ and coaches’ NK found that prior nutrition knowledge, higher levels of education, previously undertaking a nutrition course or currently majoring in nutrition studies correlated with higher NK scores. Ten of fifteen studies that assessed the effects of gender on NK stated there were no significant differences between males and females. All studies that evaluated sporting calibre and type of sport reported these had no effect on NK scores. The present study reflected these findings; previous nutrition education and higher levels of education positively affected NK score, but a relationship between score and sport played, sporting level and gender were not detected. Older participants had better GNK, which is in contrast to findings in other cohorts [32]. Of note, however, the current sample had only a small proportion of athletes from sports other than AF, and playing at the sub-elite or elite level.

## Conclusions

The findings of this study confirm that there is room for improvement in athletes’ NK. Professionals working with athletes should provide targeted advice based on nutrition topics that are poorly understood and education programs should be evaluated using validated NK measures [33]. Long SNK questionnaires are beneficial for their ability to assess gaps in NK but shorter questionnaires appear to achieve higher completion rates amongst athletes, and thus may be more practical in certain settings. The A-NSKQ is a brief tool to assess GNK and SNK that has been validated against the Rasch model and results in total (%) scores that are comparable to the NSKQ. Preliminary results using the A-NSKQ indicate that hydration, micronutrients and supplementation are topics where there may be gaps in the knowledge of recreational Australian athletes. The A-NSKQ can be used to assess the NK of athletes and inform additional data collection efforts (i.e. interviews with individual athletes), education programs, or advocate for increased nutrition education by trained professionals.

## Additional file

Additional file 1: **Table S1.** Characteristics of participants who completed the NSKQ for validation (*n* = 181). **Table S2.** Summary of RUMM2030 statistics that are assessed to determine reason for misfit to the Rash model. **Table S3.** Response and completion rates for the NSKQ and A-NSKQ. **Table S4.** Responses (percent correct) of individual items in the A-NSKQ. **Table S5.** Differences in NK on the A-NSKQ based on participant characteristics. (DOCX 34 kb)

## Abbreviations

A-NSKQ: Abridged Nutrition for Sport Knowledge Questionnaire
GNK: General nutrition knowledge
NK: Nutrition knowledge
NKQ: Nutrition knowledge questionnaire
NSKQ: Nutrition for Sport Knowledge Questionnaire
SNK: Sports nutrition knowledge

## Footnotes

1 Trakman GL, Forsyth A, Middleton K, Jenner S, Keenan A, Hoye R, Belski R. Australian football athletes lack awarenss of current sports nutrition guidelines. Int J Nutr Exerc Metab. 2017; Awaiting DOI.
