# Supplementary material for: Development and validation of a brief general and sports nutrition knowledge questionnaire and assessment of athletes’ nutrition knowledge
Source: J Int Soc Sports Nutr. 2018 Apr 19;15:17. doi: 10.1186/s12970-018-0223-1 (PMC5907737; doi:10.1186/s12970-018-0223-1)
Supplement: Supplementary file 1 — Table S1. Characteristics of participants who completed the NSKQ for validation (n = 181). Table S2. Summary of RUMM2030 statistics that are assessed to determine reason for misfit to the Rash model. Table S3. Response and completion rates for the NSKQ and A-NSKQ. Table S4. Responses (percent correct) of individual items in the A-NSKQ. Table S5. Differences in NK on the A-NSKQ based on participant characteristics. (DOCX 34 kb) [file 12970_2018_223_MOESM1_ESM.docx]

| **Table S1** Characteristics of participants who completed the NSKQ for validation (n = 181) | |  |
| --- | --- | --- |
| Characteristic | N (%) |  |
| **Gender**  *Male*  *Female* | 41 (25)  125 (75) |  |
| **Age**  *17 - 25*  *26 - 35*   1. *- 45* 2. *- 55*   *>55* | 86 (52)  40 (24)  23 (14)  9 (5)  8 (5) |  |
| **Country of Birth (COB)**  *Australia*  *Outside Australia* | 133 (81)  2 (20) |  |
| **Marital status**  *Single*  *Married/De-facto*  *Divorced* | 112 (68)  46 (28)  8 (4) |  |
| **Highest level of education**  *High school*  *Vocational training or other diploma*  *University* | 3 (2)  3 (2)  166 (96) |  |
| **Nutrition education**  Yes  No  **Plays Sports at least 1/week**  *Yes*  *No* | 97 (55)  78 (45)  100 (62)  61 (38) |  |
| 153 participants completed the questionnaire once; 28 participants completed the questionnaire on two occasions for test-retest reliability calculation. Data for all participants is presented together. Data split into first and second round completions is presented elsewhere [9]. There was data missing for gender (n=14); age (n =8); COB (n=15); marital status (n =19); education (n=9); nutrition education (n=6); sport (n=20). Percentages have been rounded to the nearest whole figure and reported based on available data. | | |

| **Table S2** Summary of RUMM2030 statistics that are assessed to determine reason for misfit to the Rash model | | |
| --- | --- | --- |
| Indicator Name | Indicator description and purpose | Interpretation |
| Individual item fit residuals | Difference between observed and expected value | >2.5 = large difference between observed and expected value |
| Item characteristic curves (ICC) | Item characteristic curve (plots person location against ‘expected value’) | Dots do not fall on the curve = problematic ICC. Indicates item is poor at discriminating between high and low scoring respondents |
| Category probability curves (CPC) | Plots ‘person location’ i.e. range in the trait being measured, against probability of endorsing a particular response | If a particular response category does not appear have the highest probability of being endorsed at least once = problematic CPC. Indicates issue with response option |
| Differential item functioning (DIF) | Assesses systematic and random differences in responses to individual items based on personal characteristics (e.g. age, gender) | A significant P-value (< 0.05 or Bonferroni adjusted value) = significant DIF |
| *Simplified and adapted from* ***[6]*** | | |

|  | | | |
| --- | --- | --- | --- |
| **Table S3** Response and completion rates for the NSKQ and A-NSKQ | | | |
| Questionnaire, data collection stage, n =number of questions | Recruitment | Response Rate | Completion Rate |
| NSKQ, stage 1 validation, n = 176 | Email to AFL Victoria community league members; posting on university student online forums; posting on sporting Facebook groups | Not known (due to use of forums and Facebook) | **44%**  **(188/462)** |
| NSKQ, stage 2 validation, n = 100 | Email to softball player league members; posting on university student online forums; posting on sporting Facebook groups | Not known (due to use of forums and Facebook) | **62%**  **(181/276)** |
| NSKQ, data collection 1, n = 89 | Elite athletes – direct contact from club dietitian | **100%** | **100%**  **(46/46)** |
| NSKQ, data collection 2, n = 89 | Recreational athletes – email to metropolitan and state leagues to forward email to players; 659 leagues emailed | **6%** of league presidents that were contacted agreed to  forward email; individual response rate not known as emails sent from 39 clubs | **56%**  **(53/94)** |
| NSKQ, data collection 3, n = 89 | Hockey players – email from club president to players (numbers unknown) | Not known; one out of 8 **(13%)** teams contacted agreed to forward link | **83%**  **(19/23)** |
| NSKQ, data collection 4, n = 89 | Soccer players – direct contact from club dietitian to 25 players | **40%** | **50%**  **(5/10)** |
| NSKQ, data collection 5, n = 89 | Lawn Bowls players – email from High Performance Manager to players (numbers unknown) | Not known; 1 club contacted | **83%**  **(10/12)** |
| NSKQ, data collection 6, n = 89 | Cricket players – email from High Performance Manager to players (number unknown) | Not known; 3 state and 3 district clubs contacted | **50%**  **(4/8)** |
| A*verage completion rate of NSKQ (excluding elite AF)* | | | 54% |
| A- NSKQ, data collection 7, n = 37 | Recreational AF players – email from club president to all players club players (3951) | **7%** | **85%**  **(177/208)** |

| **Table S4** Responses (percent correct) of individual items in the A-NSKQ | |
| --- | --- |
| **General Nutrition Knowledge** |  |
| 1. Protein eaten in excess of bodily needs can lead to fat gain | 62 |
| 1. Do you think these foods are high or low in carbohydrate? A Banana | 54 |
| 1. Do you think these foods are high or low in carbohydrate? ½ cup cooked quinoa | 61 |
| 1. Fat is required by the body to make cell membranes and molecules involved in immune function | 76 |
| 1. Do you think these foods are high or low in fat? 1/2 Cup Cottage cheese | 35 |
| 1. Do you think these foods are high or low in fat? 1 TBS Polyunsaturated margarine | 71 |
| 1. Do you think these foods are high or low in fat? 1 TBS honey | 78 |
| 1. Protein absorption in a single sitting is limited | 49 |
| 1. Do you think these foods are high or low in protein? 30g (1 ounce) Yellow Cheese | 32 |
| 1. Do you think these foods are high or low in protein? 1 Cup Baked Beans | 81 |
| 1. Do you think these foods are high or low in protein? 1/2 Cup Cooked Quinoa | 30 |
| 1. Eggs contain all the essential amino acids needed by the body | 51 |
| 1. Thiamine (Vitamin B1) is required for efficient delivery of oxygen to muscles | 6 |
| 1. Vitamins provide the body with energy (kilojoules/calories) | 59 |
| 1. When consumed as part of the diet, pure alcohol (ethanol) contains calories/kilojoules and, therefore, can lead to weight gain | 65 |
| 1. Drinking large amounts of alcohol can reduce recovery from injury | 76 |
| 1. "Binge drinking" (also referred to as heavy episodic drinking) is generally defined as: Having two or more standard alcoholic drinks on the same occasion/*Having four to five or more standard alcoholic drinks on the same occasion*/Having seven to eight or more standard alcoholic drinks on the same occasion /Not Sure | 52 |
| **Sport Nutrition Knowledge** |  |
| 1. Increasing protein in the diet is the main dietary change needed when only muscle gain is desired | 48 |
| 1. Which do you think is the best lunch option for an athlete trying to gain weight (muscle)? Assume they are training in the morning and have already had breakfast and a mid-morning snack: A 'mass gainer' protein shake and 3 - 4 scrambled eggs/Pasta with lean mincemeat and vegetable sauce, plus a dessert of fruit, yoghurt and nuts /A large piece of grilled chicken with a side salad (lettuce, cucumber, tomato)/ A large steak and fried eggs/Not Sure | 34 |
| 1. When exercising at low intensities, fat provides almost all the substrate needed to cover energy costs | 52 |
| 1. Vegetarian athletes can meet their protein requirements without the use of protein | 74 |
| 1. The protein needs of a 100 kg (220 lb) well trained resistance athlete are closest to: 75 g (2.7 ounces) per day/130 g (4.6 ounces) per day/250 g (8.8 ounces) per day/They should eat as much protein as possible/Not sure | 31 |
| 1. Athletes have increased magnesium needs due to losses in sweat | 11 |
| 1. The optimal calcium intake for athletes aged 15 to 24 years is 500 mg | 34 |
| 1. A physically fit person eating a nutritionally adequate diet can improve their performance by eating more vitamins and minerals | 13 |
| 1. Vitamin C should be routinely supplemented by athletes | 40 |
| 1. Athletes should drink water during activity in order to: Maintain sweat volume/Prevent dry mouth/Maintain plasma (blood) volume/All of the above/Not Sure | 8 |
| 1. Regarding fluid intake during physical activity, current recommendations encourage athletes to: Drink 50 - 100 ml (1.7 - 3.3 fluid ounces) every 15 - 20 minutes/Drink to a plan, based on body weight changes during training sessions performed in a similar climate/Drink sports drinks (e.g. PowerAde) instead of water when exercising/Suck on ice cubes rather than drinking during practice/Not Sure | 37 |
| 1. Before competition, athletes should aim to consume foods that are high in: Fluids, fat and carbohydrate/Fluids, fibre and carbohydrate/Fluids and carbohydrate/ Not Sure | 52 |
| 1. In events last 60 - 90 minutes, 30- 60 g (1.0 - 2.0 ounces)  of carbohydrates should be consumed per hour | 44 |
| 1. Consuming carbohydrate during exercise will assist in maintaining blood glucose levels | 58 |
| 1. Which of the following best meets the recommendations for a snack consumed during high-intensity exercise lasting around 90 minutes? A protein shake/A ripe banana /2 Boiled eggs/ A handful of nuts/Not Sure | 70 |
| 1. How much protein do you think experts recommend athletes should have after completing a resistance exercise session? 1.5g/kg body weight (~ 150 – 130 g/ 5.3 –10.6 ounces for most athletes) /1.0 g/kg body weight (~ 50 - 100 g /1.9 - 2.3 ounces) for most athletes) /0.3g/kg body weight (~ 15 - 25 g/0.53 - 0.88 ounces) for most athletes)/Not Sure | 12 |
| 1. Supplement labels may contain false or misleading information | 53 |
| 1. The purity and safety of all supplements are tested before sale | 10 |
| 1. In relation to improving sporting performance, which of the following supplements do you think has NOT been supported by a strong body of scientific evidence? Caffeine / Ferulic acid/ Bicarbonate/ Leucine/ Not Sure | 13 |
| 1. Which of the following supplements do you think is banned by the WORLD ANTI-DOPING AGENCY (WADA)? Caffeine / Bicarbonate / Carnitine/ Glycerol / Not Sure | 9 |

| **Table S5** Differences in NK on the A-NSKQ based on participant characteristics | | | | |
| --- | --- | --- | --- | --- |
| **Variable** | **Group** | **Total score , % ± SD** | **General Nutrition Knowledge, % (IQR)** | **Sports Nutrition Knowledge, % (IQR)** |
| Previous Nutrition Study | Yes (n 37)  No (n 140) | 52±11*  45±12 | 65(18)*  53(24) | 40 (10)*  35 (20) |
| Highest level of education | High school (n 85)  Diploma (n 26)  University (n 117) | 51±10*  41±13  46±12 | 65 (12)*^a^  53 (28) ^b^  59 (18) | 45 (15)* ^c^  30 (19) ^d^  36 (20) |
| Age | 17 – 25 (n 85)  26- 36 (n 66)  >36 (n 26) | 46±11  45±12  54±14 | 53 (18)*^e^  56 (24)  ^f^  65 (25) ^g^ | 40 (15)  33 (20)  35 (30) |
| Gender | M (n 69)  F (n 108) | 46±12  46±12 | 59 (18)  59 (18) | 35 (15)  30 (20) |
| Sport Played | AF (n 129)  Other (n 48) | 46±11  47±14 | 59 (18)  53 (27) | 35 (20)  40 (15) |
| Level of sport played | Metropolitan (n 144)  Other (n 33) | 46±16  46±14 | 59 (18)  53 (24) | 35 (20)  35 (20) |
| *Difference is statistically significant at P <0.005  a statistically significantly different from b (P=0.011)  c statistically significantly different from d (P=0.013)  e statistically significantly different from f (P = 0.003) and f (P= 0.004) | | | | |
